# The Effectiveness of Nutritional Strategies in the Treatment and Management of Obesity: A Systematic Review

**DOI:** 10.7759/cureus.45518

**Published:** 2023-09-19

**Authors:** Oluwakemi L Adeola, Ginikachukwu M Agudosi, Ngozi T Akueme, Okelue E Okobi, Falilatu B Akinyemi, Uchechi O Ononiwu, Henrietta S Akunne, Micheal K Akinboro, Omosefe E Ogbeifun, Matayebi Okeaya-Inneh

**Affiliations:** 1 Nutritional Sciences, Howard University, Washington, DC, USA; 2 Internal Medicine, Trinity School of Medicine, Warner Robins, USA; 3 Dermatology, University of Medical Sciences (UNIMED), Ondo, NGA; 4 Family Medicine, Larkin Community Hospital Palm Springs Campus, Miami, USA; 5 Family Medicine, Medficient Health Systems, Laurel, USA; 6 Family Medicine, Lakeside Medical Center, Belle Glade, USA; 7 Internal Medicine, Windsor University School of Medicine, Cayon, KNA; 8 Family Medicine, Imo State University College of Medicine, Alberta, CAN; 9 Psychiatry, Delta State University, Abraka, NGA; 10 Epidemiology and Biostatistics, Texas A&M Health School of Public Health, College Station, USA; 11 Public Health, University of West Florida, Pensacola, USA; 12 Family Medicine, Garki Hospital, Abuja, NGA

**Keywords:** low-protein diet, obesity treatment, low-carbohydrate diet, low-fat diet, low-energy diet, weight-loss

## Abstract

Obesity, a condition primarily resulting from positive energy balance, has become a significant global health concern. Numerous studies have demonstrated that obesity is a major risk factor for various illnesses, including different types of cancer, coronary heart disease, sleep apnea, CV stroke, type II diabetes mellitus, etc. To effectively address this issue, prevention and treatment approaches to manage body weight are crucial. There are several evidence-based approaches available for the treatment and management of obesity, taking into account factors such as body mass index classification, individual weight history, and existing comorbidities. To facilitate successful obesity treatment and management, there are pragmatic approaches and tools available, including the reduction of energy density, portion control, and diet quality enhancement. These approaches encompass the use of medications, lifestyle interventions, bariatric surgery, and formula diets. Regardless of the specific method employed, behavior change, reduction of energy intake, and increased energy expenditure are integral components for successful treatment and management of obesity. These measures allow patients to personalize and customize their dietary patterns, leading to effective and sustainable weight reduction. Incorporating physical activities and self-monitoring of individual diets are effective techniques for promoting behavior change in obesity and weight management. The main objective of this systematic review is to evaluate the effectiveness of dietary/nutritional interventions in the treatment and management of obesity through provision of valuable insights into the effectiveness of such nutritional strategies. To attain this, a comprehensive analysis of various dietary approaches and their impacts on weight will be conducted.

## Introduction and background

Obesity is a complex and largely preventable chronic condition characterized by the excessive accumulation of fat [1]. The widely accepted measure for categorizing obesity is the body mass index (BMI), calculated as weight (kg) divided by the square of height (meters) (kg/m^2^) [1]. A BMI of ≥ 25 kg/m^2^ classifies a person as overweight, while a BMI of ≥ 30 kg/m2 indicates obesity [2,3]. However, while BMI is useful for assessing obesity and being overweight, abdominal obesity, determined by waist circumference, is linked to various health risks. Therefore, medical guidelines consider both waist circumference and BMI as important tools for evaluating abdominal obesity and weight status, though they should be used in conjunction with other clinical and anthropometric parameters. Although other methods, such as MRI, dual-energy x-ray absorptiometry, and bioimpedance analysis are available for evaluating body composition and adipose tissue deposits, a number of them might be costly and others time-consuming.

The primary cause of obesity is a prolonged imbalance between energy consumption and expenditure [4-6]. Studies by the National Health and Nutrition Examination Survey (NHANES) show a significant increase in energy intake in men and women between 1971 and 2000 [7]. The rise in energy intake, without corresponding increases in energy expenditure, can lead to significant weight gain [7-9]. Studies have shown that physical activity levels have decreased over the years, contributing to weight gain [8,9]. Reduced daily occupation-linked energy expenditure in the United States has been identified as a major factor behind the observed weight gain in the adult population [10,11]. Other factors, such as genetics, environmental, and lifestyle aspects, also play a significant role in causing obesity [12].

Obesity and being overweight are now considered a global pandemic, with escalating prevalence rates in almost all countries [4,13]. Globally, 13% of adults are obese and 39% of adults are considered overweight, even as one out of every five children and adolescents is considered overweight [12-14]. By the end of 2010, obesity and being overweight were responsible for over 3.4 million deaths and a substantial loss of healthy life years globally [14]. Scholars have even predicted a decline in life expectancy due to the continuous increase in obesity rates [5]. To combat this issue, various World Health Organization (WHO) member states have adopted voluntary targets to halt the increase in obesity by 2025 [15].

Globally, obesity prevalence has doubled in over 70 countries since 1980, with the numbers continuing to rise [16-18]. If current trends persist, it is estimated that by 2030, approximately 20% of the world's adult population will be obese and 38% overweight [19]. The recent COVID-19 pandemic has also had an impact, promoting obesogenic environments due to lifestyle changes during lockdowns [20]. Consequently, the pandemic has further reinforced the ongoing obesity crisis. Therefore, there is an urgent need to focus on BMI monitoring and the implementation of evidence-based interventions to address the challenges posed by obesity.

Prevalence and impact of obesity on healthcare outcomes

Obesity prevalence has been on an upward trajectory in both the developed and developing nations across the globe and has been accompanied by an increment in the incidence rates of various obesity-related complications and chronic illnesses [21]. For instance, in the United States, obesity has become a growing challenge that has steadily increased since 1990 and affects all ethnicities and subgroups, including older persons. Thus, in the 1990s, among the beneficiaries of Medicare aged 65 years and above, obesity rates were approximated to be 13% [22], as well as 15% in the subgroup as of 2001 [23]. A number of recent studies have disclosed that between 1997 and 2006, obesity rates in the United States increased from 21% to 29% and that the rates might have been 35% [24-26].

Still, in the year 2010, obesity and being overweight were approximated to be responsible for more than 3.4 million deaths, as well as 3.9% of years of life that were lost and a further 3.8% of the disability-adjusted life years (DALYs) throughout the world [27]. Across the globe, the percentage of adult persons with BMIs of 25 and above was reported to have increased from 28.8% (28.4-29.3) in the 1980s to approximately 36.9% (36.3-37.4) in the year 2013 for men, as well as from 29.8% (29.3-30.2) in the 1980s to 38.0% (37.5-38.5) as at 2013 for the women [28]. Both the developed and developing nations have recorded considerable increments in the obesity prevalence rates, with substantial increments in prevalence rates being observed in adolescents and children in developed nations, as 23.8% of male children and adolescents and 22.6% of female children and adolescents were reported to be obese and overweight as at the end of 2013 [2]. Similarly, obesity prevalence rates in adolescents and children have been observed to steadily rise in developing nations, with increments from 8.1% to 12.9% in boys and a further increase from 8.4% to 13.4% in girls at the end of 2013. In adults, in Tonga, the approximated obesity prevalence rates surpass 50% in men and women, even as similar rates (above 50%) have been reported in women in Kiribati, Kuwait, Qatar, Libya, Samoa, Micronesia, and Tonga [2]. However, as of 2006, in developed nations, increments in the prevalence rates among adults have stabilized.

Obesity and being overweight have considerable impacts on individual health. Thus, obesity is a major risk factor for an array of chronic mental and physical conditions, such as different types of cancers, stroke, hypertension, sleep apnea, osteoarthritis, back problems, depression, various chronic mental health conditions like depression, eating and personality disorders, anxiety and psychosis-spectrum, type 2 diabetes mellitus, and hyperlipidemia, infertility, and polycystic ovarian disease among other conditions [29]. A larger proportion of the above-stated chronic physical conditions attributed to obesity might result in reduced mobility and early functional decline, which, in turn, negatively affects the regular activities of daily living (ADLs), including walking, bathing, and dressing [30,31]. As a result, obesity proffers adverse effects on the quality of life in older individuals [25,32]. According to Ogden and Flegal, obesity compromises longevity, as fewer individuals aged 75 years and above, and higher prevalence rates in individuals aged between 60 and 75 years [33].

Still, obesity and the associated chronic conditions have been linked to increased rates of hospital admissions alongside increased utilization of existing healthcare services and resources [24]. For instance, among the obese elderly persons, orthopedic procedures have been reported to be increasingly common in comparison to older persons with normal weights; an increment in weight has been linked to conditions such as osteoarthritis and increased orthopedic replacement procedure prevalence [29]. Additionally, obese persons are at elevated risk of suffering an array of health complications, prolonged hospital admission, high risk of general anesthesia, increased mortality in COVID-19 patients, and post-surgical complications attributed to increment in weight [34,35]. A number of studies have also associated the increased use of prescription drugs with severe conditions linked to obesity, including hypertension, diabetes, arthritis, and cardiovascular diseases [36].

Regarding the need for effective obesity management interventions, it can be noted that obesity has an impact on the diverse dimension of health, which makes regarded as an urgent and important public health priority, necessitating the need for comprehensive interventions and strategies for effective prevention, control, and management of the obesity epidemic [37]. However, for successful tackling of the obesity epidemic, individual level interventions are required, including dietary, lifestyle, yoga, and behavioral interventions.

Consequently, it has been noted that obesity lowers life expectancy by approximately seven years and that a 30 to 35 BMI lowers life expectancy by nearly four years even as BMIs greater than 40 reduce life expectancy by more than 10 years [38,39]. Still, obesity-related complications are either directly as a result of obesity or indirectly as a result of the mechanisms that share common causes including poor diets and sedentary lifestyles. However, the strongest association has been observed to be with type 2 diabetes, given that studies have revealed that obesity accounts for nearly 64% of type 2 diabetes in men, and a further 79% of diabetes cases in women [39]. The other notable diseases that have been associated with obesity include cardiovascular disease, stroke, hypertension, osteoarthritis, non-alcoholic fatty liver disease, venous stasis deep vein thrombosis, gastrointestinal disease, colorectal cancer, endometrial breast cancer, cholelithiasis, and gastroesophageal reflux disease, among others. Obesity remains the second major cause of cancer after smoking [22,33,39]. The obesity-related metabolic disorders include pre-diabetic state, polycystic ovary syndrome, metabolic syndrome, and hyperlipidemia. A larger proportion of individuals suffering from obstructive sleep apnea tend to be obese, even though in normal-weight individuals, the risk of obstructive sleep apnea can be attributed to the cephalometric defects because of diseases like tonsillo-adenoid hypertrophy in children [40].

Research objectives

Although several research studies focusing on obesity management have been published to date, only a limited amount of evidence exists with regard to compliance with healthy diets to maintain healthy body weights, as well as manage and treat obesity. Furthermore, with a vast amount of existing evidence being drawn from mostly cross-sectional research studies, drawing conclusion regarding the effects of different nutritional interventions on the management and treatment of obesity is very difficult. Numerous dietary patterns, both macronutrient and food-based, can lead to weight loss. A key strategy for weight management that can be applied across dietary patterns is to reduce energy density. Clinical trials show that reducing energy density is effective for weight loss and weight loss maintenance. A variety of practical strategies and tools can help facilitate successful weight management by reducing energy density, providing portion control, and improving diet quality. The flexibility of energy density gives patients options to tailor and personalize their dietary patterns to reduce energy intake for sustainable weight loss. To our knowledge, at present, a limited number of systematic reviews and meta-analysis have synthesized the effects of nutrition/diet interventions on obesity management and treatment. Thus, an enhanced comprehension of the effects of nutrition and diets on obesity management and treatment is prone to facilitate their execution in a bit to reduce the prevalence of obesity. As such, the objective of this systematic review entails reviewing the role of diet and nutrition in the treatment and management of obesity.

## Review

Methodology and materials

To gather pertinent research and peer-reviewed articles published in English language, an in-depth search was conducted on online medical databases including Embase, PubMed, Web of Science, and SCOPUS, as well as Google Scholar, up to June 2023. The articles selected included health assessment studies and epidemiological studies that comprised anonymized data and various multi-center studies, as well as published review articles. Duplicate data sources were also identified through a comparison of studies and articles from similar population years, and study sources with more valid details were selected and utilized. For the literature search, keywords that included weight loss, obesity treatment, low-energy diet, low-fat diet, low-carbohydrate diet, and low-protein diet were used. The literature search yielded a total of 1238 articles. 

Inclusion and Exclusion Criteria

Following the removal of all duplicates, pertinent articles were chosen in three distinct phases. The initial phase entailed screening of the articles' titles and abstracts while the second phase entailed the exclusion of articles considered irrelevant. The final phase entailed an in-depth full-text exploration of the recruited papers with the objective of selecting only pertinent articles. The three article screening phases were conducted by three independent reviewers, and the discrepancies realized were solved through consensus and consultations.

The inclusion criteria included original studies, including randomized controlled trials, crossover design studies, and prospective cohort studies that satisfied the following criteria: studies that focused on nutritional management and treatment of obesity; studies on obese and overweight persons; dietary interventions; published in English language; and conducted in the last 10 years. Furthermore, editorials, sponsored clinical trials, and narrative reviews were excluded. The abstracts of the articles were initially evaluated leading to the removal of 775 articles.

For this systematic review, important data were extracted from the eligible articles as follows: (a) General study characteristics, including the authors’ names, year of study, year of publication, and sampling methods employed, (b) the study population characteristics, including race, sample size, gender and age of study participants, and follow-up, (c) intervention type and duration, as well as the measures utilized in weight assessment, and (d) the main study findings.

**Figure 1:**
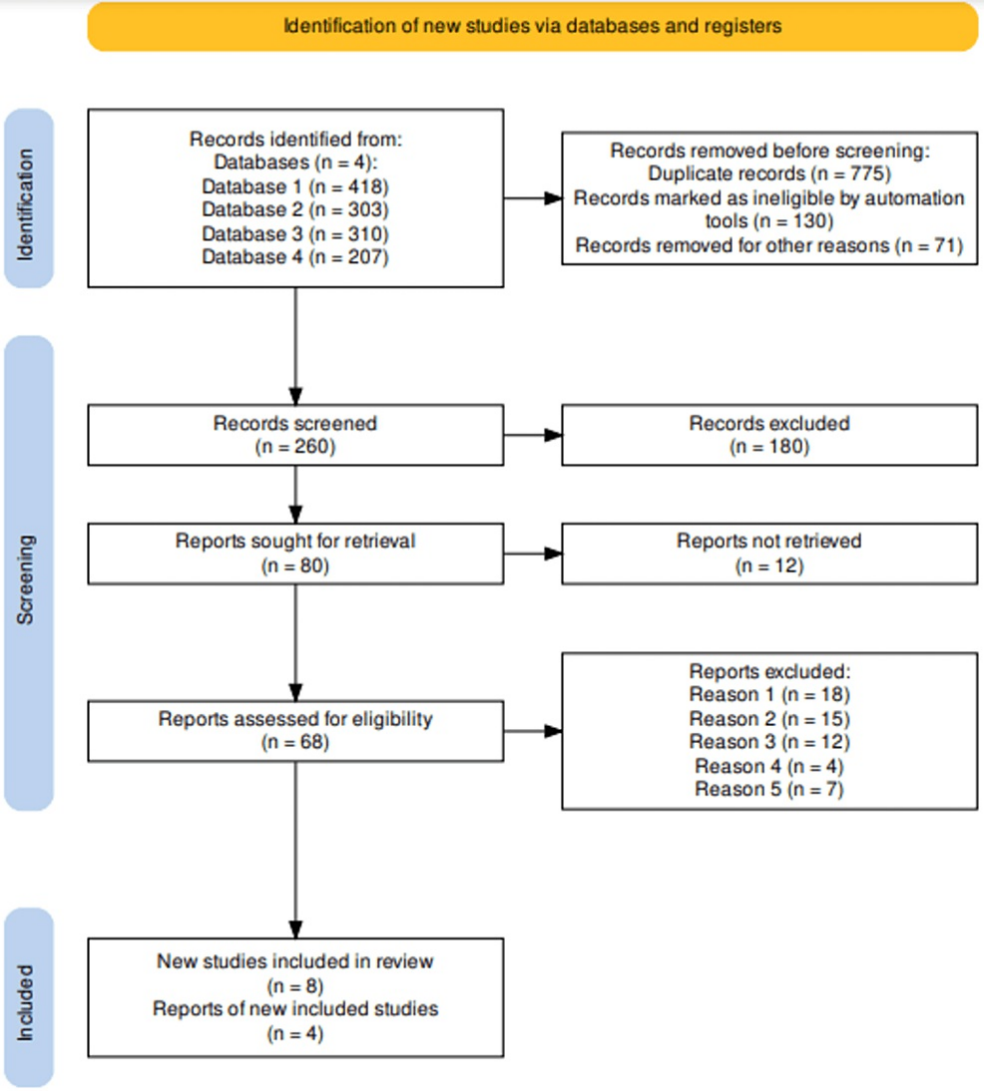
PRISMA flow for selected studies PRISMA flow diagram indicating the study selection process for the studies included in this systematic review. PRISMA: Preferred Reporting Items for Systematic Reviews and Meta-Analyses

Furthermore, for this systematic review, the selection process was conducted using Preferred Reporting Items for Systematic Reviews and Meta-Analyses (PRISMA). Thus, from the study selection, a total of 1238 article records were retrieved from the in-depth database search conducted. The articles were screened leading to the removal of 775 duplicates alongside 130 articles that were found to be ineligible by automation, and additional 71 records that were excluded for other reasons (We excluded studies that did not align with our study's objectives, as well as animal studies. Additionally, we excluded studies published in non-peer-reviewed journals and dissertations. Studies originally published in languages other than English were also excluded. Furthermore, we did not incorporate opinion pieces, articles not authored by academics, secondary studies, scoping reviews, or any other types of research that did not qualify as primary studies). As a result, a total of 260 eligible articles were screened and a further 180 were excluded. The remaining 80 articles were sought for retrieval, out of which 12 articles were not retrieved. Therefore, a total of 68 articles were evaluated for eligibility resulting in the exclusion of 22 articles after full-text screening for the following reasons: protocol (18 articles); full text missing after reaching out to the authors (15 articles); preprint (12 articles); failure to report limitations (four articles); and failure to investigate the targeted intervention (seven articles). The complete article selection process has been presented in the PRISMA flow diagram in Figure 1.

In accordance with the PRISMA system above, we have incorporated the selected studies into Table 1, and also in the reference section [1-80].

**Table 1:** A list of studies selected and included in this systematic review A table indicating the list of studies included in this systematic review, showing the authors' names, study titles, study objectives, study design, sample size, and study findings.

Author	Study	Study Objectives	Design	Sample Size	Main findings
Hannon et al. [50]	Lower energy-dense ready meal consumption affects self-reported appetite ratings with no effect on subsequent food intake in women.	To compare commercially available equicaloric ready meals differing in energy density on satiety and food intake.	Randomized Control crossover study	Twenty-six female participants aged 18-65 years; body mass index of 28.8 ± 3.0 kg/m^2^	The participants felt more satiated after consuming ready meals of the same energy content but larger portion size. Despite no significant difference in short-term EI between the ready meals, the results indicated that the LEDRM produced beneficial subjective satiety responses and, therefore, can help to improve the nutritional content of meals i.e., reduce saturated fat consumption.
Bottone et al. [52]	Obese older adults report high satisfaction and positive experiences with care.	To examine the independent impact of obesity on patient satisfaction and experiences with care in adults 65 years of age and older with Medigap insurance.	Mixed method research	Fifty-three thousand two hundred and eighty six randomly chosen adults with an AARP Medicare Supplement Insurance Plan	Relative to normal weight, obesity was significantly associated with higher patient satisfaction and better experiences with care in seven of the eight ratings measured.
Champagne et al. [56]	Fiber intake, dietary energy density, and diet-type predict 6-month weight-loss in free-living adults who adhered to prescribed macronutrient and energy composition of varying diets.	To identify predictors of weight loss after six months in participants who adhered to energy and macronutrient assignments.	Mixed methods research	Three hundred and forty-five participants	The decrease in energy density was positively associated with weight change for each diet-type; however, this effect was most profound in the high-fat, average-protein diet, suggesting that dietary factors may positively influence weight loss in addition to energy intake and macronutrient composition.
Fung et al. [57]	Low-carbohydrate diets and all-cause and cause-specific mortality: two cohort studies.	To provide a mechanistic paradigm for future research in the area of ACM.	Prospective cohort study	Eighty-five thousand three hundred and sixty-eight women (aged 34-59 years at baseline) and 44,548 men (aged 40-75 years at baseline) without heart disease, cancer, or diabetes.	To examine the association of low-carbohydrate diets with mortality during 26 years of follow-up in women and 20 years in men.
Lowe et al. [62]	Meal replacements, reduced energy density eating, and weight loss maintenance in primary care patients: a randomized controlled trial.	To compare the absence of presence of meal replacements (MRs) alongside energy density (ED) intervention in facilitating maintenance of weight loss.	Mixed method research	Two hundred and thirty-eight overweight patients with mean BMI = 39.5 kg/m^2^	Each group with the exception of ED+/MR- regained substantial weight during the follow-up, while the ED+/MR- group was observed to have regained significantly less weight compared to the control group during follow-up assessments.
Flood et al. [64]	The effects of beverage type and portion size on beverage consumption and lunch intake.	To examine the impact of increasing beverage portion size on beverage and food intake.	Crossover design	Thirty-three subjects	Increasing beverage portion size significantly increased the weight of beverage consumed, regardless of the type of beverage served (P < 0.05). As a consequence, for the caloric beverage, energy intake from the beverage increased by 10% for women and 26% for men when there was a 50% increase in the portion served (P < 0.01).
Sacks et al. [67]	Comparison of weight-loss diets with different compositions of fat, protein, and carbohydrates.	To assess the possible advantage for weight loss of a diet that emphasizes protein, fat, or carbohydrates	Randomized control trial	Eight hundred and eleven overweight adults.	Reduced-calorie diets result in clinically meaningful weight loss regardless of which macronutrients they emphasize.
Shai et al. [68]	Weight loss with a low-carbohydrate, Mediterranean, or low-fat diet	To compare the effectiveness and safety of three nutritional protocols: a low-fat, restricted-calorie diet; a Mediterranean, restricted-calorie diet; and a low-carbohydrate, non–restricted-calorie diet.	Dietary Intervention Randomized Controlled Trial (DIRECT)	Three hundred and twenty-two moderately obese participants	Mediterranean and low-carbohydrate diets may be effective alternatives to low-fat diets. The more favorable effects on lipids (with the low-carbohydrate diet) and on glycemic control (with the Mediterranean diet) suggest that personal preferences and metabolic considerations might inform individualized tailoring of dietary interventions.
Gardner et al. [69]	Effect of Low-Fat vs Low-Carbohydrate Diet on 12-Month Weight Loss in Overweight Adults and the Association With Genotype Pattern or Insulin Secretion: The DIETFITS Randomized Clinical Trial.	To determine the effect of a healthy low-fat (HLF) diet vs a healthy low-carbohydrate (HLC) diet on weight change and if genotype pattern or insulin secretion are related to the dietary effects on weight loss.	Diet Intervention Examining The Factors Interacting with Treatment Success (DIETFITS) randomized clinical trial	Six hundred and nine adult participants aged 18 to 50 years with a BMI of between 28 and 40.	There was no significant difference in weight change between a healthy low-fat diet vs a healthy low-carbohydrate diet, and neither genotype pattern nor baseline insulin secretion was associated with the dietary effects on weight loss.
Jenkins et al. [70]	The effect of a plant-based low-carbohydrate (“Eco-Atkins”) diet on body weight and blood lipid concentrations in hyperlipidemic subjects	To examine the effect of a plant-based low-carbohydrate ("Eco-Atkins") diet on body weight and blood lipid concentrations in hyperlipidemic subjects.	Randomized controlled trial	Hyperlipidemic subjects	The plant-based low-carbohydrate ("Eco-Atkins") diet resulted in significant reductions in body weight and improvements in blood lipid concentrations, particularly a decrease in LDL cholesterol and triglycerides. HDL cholesterol levels remained stable or slightly increased.
Esposito et al. [72]	Mediterranean diet and weight loss: Meta-analysis of randomized controlled trials.	To assess the effects of Mediterranean diets on weight loss and obesity management	Meta-analysis of randomized controlled trials.	Three thousand four hundred and thirty-six participants (1,848 assigned to a Mediterranean diet and 1,588 assigned to a control diet)	The effect of Mediterranean diet on body weight was greater in association with energy restriction (mean difference, -3.88 kg, -6.54 to -1.21 kg), increased physical activity (-4.01 kg, -5.79 to -2.23 kg), and follow up longer than six months (-2.69 kg, -3.99 to -1.38 kg).
Willett and Leibel [80]	Dietary fat is not a major determinant of body fat.	To evaluate the effect of dietary fat on adiposity	Randomized controlled trials	Two hundred and four participants	In the short-term, modest reductions in body weight are typically seen in individuals randomized to diets with a lower percentage of calories from fat.

Discussion

The comprehension of the biology that underlies food intake and weight management is exceptionally pertinent to the treatment and management of obesity and is a major requirement in the development of effective weight loss interventions and strategies. Dietary intake manipulation with the objective of influencing the outcomes of obesity has been central to obesity treatment and management for several centuries. Among the core tenets of obesity prevention and weight loss interventions is energy intake restriction. Thus, healthcare practitioners and dietitians have over the years prescribed energy-limited diets as the initial line therapy for obesity, and this has also been proposed by several dietary guidelines and scientific societies. The restricted energy diets tend to adhere to the golden rule, which maintains that reducing the everyday energy intake by approximately 500 kilocalories is likely to result in a weight loss of nearly 0.5 kilograms per week, which translates to nearly two kilograms every month [41]. The above observations have been corroborated by the National Institute of Health (NIH) obesity treatment guidelines that propose that class 1 obese persons with two or increased amount of risk factors need to reduce their energy consumption by 500 k/calories every day and that class II obese persons need to reduce their energy consumption by between 500 and 1000 kilo calories every day to realize a 2 kg weight loss per month. Nevertheless, such reduction in calories ingested is mainly attained through the control of the size of portions eaten, lowering the quantity of saturated fats, total fats, and carbohydrates ingested while also increasing the amount of fiber from vegetables, fruits, and proteins, with the objective of reducing the overall energy density in the diet even as the satiating effects are improved [42].

Regardless of the observed general agreement with regard to the soundness underlying such interventions, findings of long-term research studies have disclosed a modest efficiency at best, an increasingly varied response, alongside low lost weight maintenance [43]. In the meta-analysis conducted by Anderson et al., which evaluated a total of 29 long-term clinical trials focusing on weight maintenance, the researchers disclosed that after a five-year period, only 3% of the weight loss was maintained [44]. The increasingly high rate of failure to successfully maintain weight loss, approximated to be over 80%, has been attributed to aspects that include metabolic adaptations and different compensatory mechanisms, which are known to maintain the body energy stores while also defending the body weight [45,46]. To this end, extensive studies have been conducted on the metabolic responses to weight loss and energy consumption restrictions, even though some aspects of it have remained elusive. The upregulation of the ghrelin hunger hormone and cortisol levels, alongside considerable reduction of testosterone, anorexigenic hormones that include CCK, insulin, leptin, PYY and GLP-1, various thyroid hormones and energy expenditure reduction by nearly 28%, and amplified perception of the foods reward values are some of the notable compensatory mechanisms following weight loss, enhancing energy consumption and making the dieters susceptible to weight loss maintenance failure [47]. Such changes in the profile of the endocrine have been reported to persist past the weight loss period and for at least one year.

Nevertheless, divergent nutritional interventions, approaches, and strategies have been developed and suggested to directly influence the response system through exploitation of the system’s sensitivity to diet composition in several ways and on numerous fronts. Such interventions and approaches have been broadly classified into macronutrient focused interventions, which include high protein, low carbohydrate (carbohydrates are not mandatory to burn fats, but they can influence the way your body uses fats for energy. The relationship between carbohydrates and fat metabolism is complex and depends on several factors, including your diet, activity level, and overall health) and low-fat diet and highlights the diverse macronutrients’ contributions to energy homeostasis and metabolism; dietary focused interventions, which includes the Mediterranean diets, as well as dietary timing focused intervention, which include intermittent fasting [48-53].

Previous studies revealed that the consumption of low-energy dense diets such as fish, fruits, vegetables, and lean meats might not just reduce the sense of hunger but additionally reduce the consumption of energy, and, as a result, assisting in weight loss [49]. For instance, a cross-over study conducted by Hannon et al. compared the influence of high-energy dense ready meals (HEDRM) to the low-energy dense ready mills (LEDRM) with regard to food intake and satiety [50]. The study disclosed that despite the LEDRM not realizing a lower energy intake, it not only increased fullness but also aided in enhancing the meals’ nutritional contents while simultaneously decreasing saturated fats consumption [47-53].

Additionally, this systematic review has acknowledged that broader inter-individual differences exist in weight loss in relation to dietary interventions. The differences in response may be mitigated through the identifying factors modifying effects of specific dietary intervention. For instance, evidence drawn from sub-group evaluations indicate that weight loss response on either low or high carbohydrate diets is linked to insulin sensitivity, and enhanced response has been observed in a low-carbohydrate diet compared to high-carbohydrate diets in individuals who are insulin-resistant, as opposed to those who are insulin-sensitive [51]. Regardless of the above findings, a larger proportion of existing literature has disclosed that the average weight loss response to a broader array of dietary macronutrient along with other forms of dietary manipulations are comparable in addition to being a function of adherence and energy limitations realized [52]. A pertinent instance based on the concept of popularization entails the utilization of low-glycemic index diets or the low-glycemic load diets for weight reduction. Thus, low-glycemic load diets have been acknowledged to generate lesser and increasingly progressive increment in the levels of blood glucose, resulting in reduction in the insulin secretion stimulation [23]. A recent Comprehensive Assessment of Long Term Effects of Reducing Intake of Energy (CALERIE) trial evaluated the weight loss effects of high-glycemic load diets in comparison to the low-glycemic load diets within the contexts of 30% calorie limitation [53]. Following a six-month feeding duration, the two participant groups were tasked with self-administration of the allocated dietary program for another six months. However, at 12 months, there was no statistically significant difference with regard to weight loss for the two groups, as the high-glycemic load diet group attained 8% weight loss while the low-glycemic load diet group attained 7.8% weight loss [53]. As such, it is recommended that prospective research studies should tackle the issue of variable responses within the contexts of diverse nutrition profiles and diet compositions, and in dissimilar biological and genetic makeups.

Interpretation of findings and comparison with findings of previous studies

Dietary intervention forms the cornerstone of obesity management and treatment, as well as any weight loss therapy. A larger proportion of existing dietary regimens recommended for weight loss lay emphasis on macronutrient compositions and energy content. The effectiveness of a dietary regimen is determined by its energy content. According to NIH obesity treatment guidelines, people with class I obesity and two or more risk factors should reduce their daily energy intake by 500 calories [54]. For individuals with class II and class III obesity, the recommendation is to reduce daily energy intake by 500 to 1000 calories to achieve a weekly weight loss of 0.5 kg. In order to offer diets that lead to attainment of the preferred energy deficits, Mourao maintains that it is critical to establish the daily energy needs of the obese person, and this may be assess through the use of the WHO equation, Harris-Benedict equation, and the American Gastroenterological Association dietary guideline [55]. Thus, nutrition has been touted to play a significant and vital role in obesity treatment and management, as it aids in the management of calorie demands through the consumption of healthy diets with appropriate energy intakes that are not linked to obesity. As such, the diet energy consumption, including interventions such as intermittent fasting (Intermittent fasting has been associated with the stimulation of autophagy and a potentially reduced risk of cancer), weight loss programs, personalized nutrition, and macronutrients are important in obesity treatment and management [48-53].

Various studies have concluded that altering the proportions of macronutrients consumed is prone to result in weight loss [56]. However, other studies have indicated that the macronutrient composition of the diets are not pertinent to obesity treatment and management but rather the macronutrients’ energy content [57,58]. In agreement, Smethers and Rolls maintain that, as carbohydrates, lipids, and proteins have dissimilar impacts on energy metabolism, satiety, and appetite, it is instinctual for one to consider that changing the diets’ macronutrients proportion with comparable overall amount of calories will result in weight loss along with changes in the body composition [59]. Moreover, a diet’s energy density might be altered in isocaloric diets, leading to differences in macronutrient composition. Given the restricted aptitude for storage of carbohydrates and proteins within the body, as well as the almost infinite ability to store fats, the human body has the aptitude to effectively and acutely regulate the carbohydrate and protein balance [57]. How the contents of the macronutrient diet affect the balance of the body energy is, to a certain degree, dependent on the body’s energy state, including the neutral, positive, or negative energy balance. In this regard, the controlled feeding trials conducted by Stelmach-Mardas et al. and Greene et al. disclosed that there was no considerable difference in relation to weight loss when the diets’ carbohydrate or fat contents were reduced, as long as comparable total energy reductions exist [60,61]. On the contrary, in their study, Smethers et al. observed that during ad libitum consumption, considerable differences in weight loss outcomes were observed between the low-fat diets and high-fat diets [59]. The observed difference was attributed to the high-fat diet induced thermogenesis, along with the low energy consumption associated with proteins and carbohydrates in comparison to fats [61].

The manipulation of the levels of dietary protein (35% of the energy in form of protein) is a preferred dietary intervention with regard to obesity treatment and management, as it aids in weight loss and weight loss maintenance. Diets consisting of high protein have been acknowledged to enhance diet-induced thermogenesis, in addition to reducing the energy consumption by changing the hormones responsible for satiety, all of which are responsible for the promotion of negative balances [62,63]. In agreement with this finding, a number of randomized controlled trials (RCTs) and ecological studies have disclosed that the high-protein diets proffer favorable outcomes in relation to weight management [64]. Nonetheless, clinically significant weight loss might take place across a wider array of macronutrient compositions, especially the fluctuating proportions of fats and carbohydrates that tend to vary between the dietary proposals and claims [54]. The above observation has been corroborated by findings of the study that evaluated four diets with diverse content of fat that ranged from 20% to 40%, protein content ranging from 15% to 25%, and carbohydrate content ranging from 35% to 65%, which disclosed that there was comparable weight loss between the diverse dietary interventions over the study period of two years [65]. No considerable dissimilarities were noted in satiety and hunger ratings for the four diets evaluated.

With regard to the efficiency of the different macronutrients in the management and treatment of obesity, a meta-analysis involving 16 trials disclosed that consumption of low-fat for a period of 2 to 12 months resulted in an average weight loss of 3.2 kilograms, in addition to improving the risk factors for cardiovascular disease [66]. Further, Sacks conducted a two-year randomized controlled study with 811 participants put into four diet groups with diverse energy consumption from carbohydrates, proteins, and fats (35, 25, and 40%; 54, 14, and 40%; 55, 25, and 20%, and 65, 15, and 20%, respectively [67]. Following two years of the dietary intervention, the findings disclosed that a weight loss of nearly four kilograms was realized and that the different groups did not register any considerable differences [67]. Additionally, a study comparing three distinct dietary interventions, including low-fat/low-energy diet, low-carbohydrate/non-energy reduced diet, and Mediterranean/low-energy diet, disclosed comparable findings [68]. Thus, after the two-year dietary intervention period, the weight loss realized was 5.5, 4.6, and 3.3 kg, correspondingly [68]. Further, a study conducted by Gardner et al. and with 609 with 28 BMI and 40kg/m^2^, the average weight loss realized was nearly 5.3 kg for the low-fat diet and 6.0kg for the low-carbohydrate diet after a 1 year of the dietary intervention [69]. Additionally, the studies on consumption of various plant-based types of Mediterranean or Atkins diets revealed achievement of modest weight loss [68,70,71], even as a recent meta-analysis showed that low-energy Mediterranean diets resulted in moderate weight loss [72].

Additionally, in their study, Andela et al. reviewed the “efficacy of very low-energy diet interventions for weight loss” and disclosed that weight-linked outcomes registered considerable improvements post-intervention in all research studies that were reviewed [73]. Thus, the meta-analysis of the 20 research studies disclosed an average of 10.1kg weight loss after the dietary interventions that lasted between 3 and 20 weeks. Further, the moderator analysis conducted on the findings of the 20 studies indicated increased weight loss in studies that focused on adolescents aged between 10 and 18 years, in addition to developing diet replacement interventions [55]. Still, a meta-analysis of seven studies reported the weight during follow-up, which was between 5 and 14.5 months, to be 5.3 kilograms average weight loss. Even though there were limited details regarding the negative events, the findings of the study indicated that low-energy diets intervention programs were effective in the treatment and management of obesity.

Regarding the low-carbohydrate diets as interventions for obesity, it can be noted that a diet’s carbohydrate content is a vital determining factor of the short-term (below two weeks) weight loss outcomes. Both very low carbohydrate (0 - < 60 grams) and low-carbohydrate (60-150 grams) have become increasingly popular over the years. The utilization of glycogen takes place in instances where the consumption of carbohydrates is limited. Thus, in instances where the consumption of carbohydrates is below 50 grams per day, ketosis is prone to develop from glycogenolysis, which, in turn, leads to fluid loss. According to Chao, a larger proportion of existing low-carbohydrate diets, including the Atkins diet, tend to restrict the consumption of carbohydrates to 20 grams per day, even as they permit unlimited intake of amounts of proteins and fats [54]. A recent meta-analysis of five studies disclosed that weight loss at six months that favored a low-carbohydrate diet over a low-fat diet could not be sustained at 12 months. High-density lipoprotein cholesterol alongside triglycerides have been observed to change increasingly favorably in individuals on low-fat diets [57]. Consequently, the very low-carbon diets (VLCDs) have energy contents ranging between 200 and 800 kilocalories per day. As such, diets with less than 200 kilocalories per day are considered starvation diets [21]. Generally, the VLCDs are not prescribed for universal usage, given that they proffer considerable adverse effects that include electrolyte imbalance, increased risk of developing gallstones, and reduction in blood pressure [74]. Therefore, the use of VCLDs must be closely monitored by a medical expert.

Based on these systematic review findings, every kind of diet recommended for obesity treatment and management has individual proponents. A meta-analysis comprising 80 weight loss and obesity treatment studies recorded a weight loss of between 5 and 8.5 kg (5 to 9%), which was noted in the initial six months from the different interventions that entailed energy diet reduction and weight loss therapies with weight plateaus at nearly six months, and with 3 to 6k (6%) weight loss maintenance at 48 months [75]. Several experiments are investigating the effects of macronutrient distribution in energy-restricted diets on excessive body weight management. Very-low-energy diets (1670 to 3350 kJ or 400 to 800 kcal per day) show short-term benefits, leading to a daily weight reduction of 300 to 500 grams, prioritizing energy restriction over macronutrient composition. Low-carb or low-fat moderately energy-restricted diets typically result in a weekly weight loss of 0.5 to 1.0 kg, while high-protein diets lead to losses of 0.2 to 0.4 kg per week in weight-reduction programs [76]. On the contrary, all the studies reviewed in this systematic review disclosed that adherence to prescribed diets was a vital determinant of weight loss and obesity management and treatment. As such, selecting diets with macronutrient components based on aspects that include the taste preference of the subject may result in the attainment of improved compliance and effective obesity treatment and management [66,77].

Additionally, a study was conducted by the National Institutes of Health and entailed a review of 34 RCTs with the objective of evaluating the efficiency of low-energy diets in reducing the body weight, reducing abdominal fats, and cardiorespiratory health [78]. The study disclosed that the low-energy diets were capable of significantly reducing the overall body weight by an average of approximately 8% within a period of between 3 and 12 months. Further, the study disclosed that interventions focusing on weight loss and maintenance that lasted between 3 and 4.5 years, which was found in four of the RCTs reviewed, led to about 4% average weight loss, which was lower compared to the proposed description of successful weight loss (a 10% decrement in body weight sustained for over 12 months) [79]. The findings of this systematic review indicate that low-energy diets do not only lead to weight loss but also reductions in abdominal fats, as indicated by the decreased in the circumference of the waist by between 1.5 cm and 9.5 cm.

The study conducted by the National Institutes of Health additionally disclosed that low-energy diets only do not enhance cardiorespiratory health as assessed through the maximum oxygen intake rate [78], and this reinforces the significance of combining dietary interventions with physical exercise for weight-loss. Dietary interventions, including consumption of low-energy diets, alongside behavioral therapy have been found to bring about extra weight loss in the short-run (12 months) as opposed to the long-run (36 to 60 months) [78].

At present, an ongoing discourse exists with regard to the efficiency of low-fat diets in weight loss [80,81]. Astrup et al. carried out an in-depth meta-analysis involving 16 studies conducted over a period of 2 to 12 months, and out of which 14 studies were RCTs [82]. The study disclosed that low-fat diets devoid of deliberate energy intake restriction led to greater weight loss at 3.5 kg, compared to the habitual and medium-fat diets ad libitum [82]. The study registered considerable weight loss in much heavier participants. As such, Astrup et al. concluded that despite small achievement of weight loss with the dietary interventions involving consumption of low-fat energy diets, the view that it was realized with reductions in the amounts of dietary fats without planned energy consumption reductions might be of immense interest from the perspective of public health [82]. Still, in their respective studies, Yu-Poth et al. and Hooper et al. observed that decrements in fat consumption was directly linked to weight loss [83,84]. Thus, in the meta-analysis conducted by Yu-Poth et al. involving 37 trials that utilized National Cholesterol Education Program (NCEP) Step I and II diets, in which each 1% reduction in energy intake was linked to approximately 0.28 kilograms weight loss (R2 = 0.57, p < 0.0001) [83,84]. Consequently, a meta-analysis of six RCTs focusing on weight loss, Pirozzo et al. disclosed that there were no considerable differences with regard to the effects of low-fat diets along with other weight loss diets on individuals with obesity and overweightness at between 6 and 18 months [81]. In all the trials, an overall weight loss of 2 to 4 kg was realized between 12 and 18 months. A review by the National Institute of Health also disclosed that low-fat diets comprise 20-30% of the overall energy consumption; minimal evidence shows that low-fat diets without energy consumption leads to weight loss exist [78,85,86]. This observation has been further corroborated by the study conducted by Foster-Schubert et al. focusing on obese female participants, comparing the results of a diet-based intervention offering 5040 kJ/day and comprising four dissimilar fat contents, including 10%, 20%, 30%, and 40% [85]. The study findings disclosed that there were no statistically significant differences with regard to weight loss after three months, as the weight loss realized was -4.5kg, -6.8kg, -6.9kg, and -6.8kg for the 10%, 20%, 30%, and 40% fat content groups/categories.

Practical implications

This systematic literature review has disclosed that, in the short-term, the consumption of low-energy diets, including vegetables, low fats, low-proteins, and fruits, reduces overall energy consumption, which, in turn, leads to considerable weight loss in obese and overweight persons. Nevertheless, regardless of such findings, more recent evidence indicates that the mean weight loss response to a broad array of dietary macronutrients along with dietary manipulations are comparable and are as a result of adherence to the dietary intervention and achievement of energy restriction. Eventually, in the future, it is projected that obesity interventions will entail the prescription of personalized nutrient profiles with the objective of matching the individual’s specific requirements. Thus, the inter-personal response to certain diets and foods enables the development of the necessary opportunity for designing of prescriptions capable of leading to the realization of optimized outcomes in comparison to the standard general guidelines. The personalization targets might take in definite dietary patters, including reduction in sugar consumption and low glycemic load; the exclusion of specific nutrients in the diets, including gluten; diet supplements; microbiome nutritional alteration, and consideration of different biological factors that include insulin sensitivity levels. As the targets involved in energy homeostasis vary considerably, the necessary keys for unlocking the energy balance and nutrient environment interactions might eventually include proteomics, metabolomics, and genotyping to effectively direct the nutrient intervention and therapy. Nevertheless, at present, the concepts are mainly within the domains of prospective use as technology and research advance. Presently, the management of energy balance, which can only be attained through effective nutritional interventions, is the most promising and appropriate nutrition consideration.

Complications of weight loss

Some studies have suggested that prolonged focus on weight, extreme dieting, and rapid weight loss can contribute to psychological issues such as eating disorders, body image concerns, and a preoccupation with food. Also, losing a considerable amount of weight due to dieting or an eating disorder may cause thyroid gland fluctuations, resulting in reduced levels of these reproductive hormones, secondary amenorrhea, or an absence of menstruation. In addition, chronic fixations on weight loss without proper caloric and nutritional monitoring, drastic calorie restriction, or unbalanced diets can lead to nutrient deficiencies, causing problems such as anemia, weakened immune function, and bone diseases such as osteoporosis. Also, severe calorie restriction or excessive exercise can result in electrolyte imbalances, leading to some disorders associated with electrolyte imbalance, such as muscle cramps, irregular heartbeats, or even seizures in extreme cases. Rapid weight loss can increase the risk of developing gallstones. The stress related to extreme weight loss has also been reported to be related to stress-induced alopecia. Other indirect disadvantages of weight loss may include complications of procedures adopted for weight loss, such as bariatric surgeries, for example, surgical and post-surgical complications, poor wound healing, and refeeding syndrome. However, due to the scarcity of data on the adverse effects of acute and chronic weight loss, further studies are needed to evaluate short and long-term complications related to acute or chronic weight loss [10,43,50, 71-73, 87-91].

Strengths and limitations

Among the notable strengths of this systematic review entails the observation that it was carried out based on the Cochrane guidance to ascertain that the methodology was not only robust but also systematic. Further, for this systematic review, the scope was clear with aptly pre-defined inclusion and exclusion criteria for the literature used, as well as the findings and study design. Still, a comprehensive and systematic literature search was carried out with an aptly predefined literature search strategy without language restrictions in several databases. The search strategy reporting followed the PRISMA statement requirements.

Consequently, for this systematic review, a number of limitations were noted including the use of observational research design, which is subject to random and systematic errors. Such systematic errors include the increased risk of selection bias, selective outcome reporting, insufficient blinding, and attrition bias, among others. The other notable limitation noted regarded the heterogeneity in the sample populations and sizes in the different studies reviewed. Thus, the different studies reviewed focused on different sample populations with regard to gender, age, race, and ethnicities, and this might have had considerable impacts on the studies’ outcomes.

## Conclusions

In conclusion, understanding the biology of food intake and weight management is crucial for effective obesity treatment and the development of weight loss strategies. Also, dietary manipulation, particularly energy intake restriction, has long been central to obesity treatment and management, with guidelines recommending calorie reductions of 500 to 1000 kilocalories per day. Lastly, while various dietary approaches have been explored, including macronutrient-focused interventions and low-energy diets, long-term studies show modest and variable effectiveness, with factors like metabolic adaptations contributing to weight loss maintenance challenges. Personalized nutrition may play a role in future obesity interventions. Additionally, studies that test the impact of tailoring choice of dietary interventions to the individual’s ability to adhere long term are needed. Understanding the physiologic response to weight reduction might enable the field to define better dietary methods of caloric restriction during weight reduction and maintenance.
